# Characterization of Milk Fat Globule Membrane Phospholipids in Colostrum of *Holstein cows*, *Yaks* and *Buffaloes* as Well as in *Yak* Colostrum and Mature Milk

**DOI:** 10.3390/foods15020317

**Published:** 2026-01-15

**Authors:** Jie Luo, Yu Cao, Hui Zhou, Fangfang Yan, Shan Wu, Hao Zhang, Xiankang Fan

**Affiliations:** 1 College of Food Science and Technology, Hunan Agricultural University, Changsha 410128, China; luojie@hunau.edu.cn (J.L.); 17373499011@163.com (Y.C.); paradise917@163.com (H.Z.); yanfangfang@hunau.edu.cn (F.Y.); 2 Inner Mongolia Enterprise Key Laboratory of Dairy Nutrition, Health & Safety, Inner Mongolia Mengniu Dairy (Group) Co., Ltd., Hohhot 011500, China; wushan331314@163.com; 3 College of Food Science and Nutritional Engineering, China Agricultural University, Beijing 100083, China

**Keywords:** milk fat globule membrane, lipid, phospholipid, *Yak* milk, whole-target lipidomics

## Abstract

Milk fat globule membrane (MFGM) phospholipids could promote the development of infants’ brain, nervous system and digestive system. This research conducted a comparative analysis of phospholipid composition in MFGM of colostrum from different bovine species (*Holstein cattle*, *yak*, and *Buffalo*), with a particular focus on analyzing phospholipid variations in *yak* MFGM across different lactation stages. Chromatographic quantification revealed phosphatidylcholine (PC) as the predominant phospholipid class (34.7–47.44%) in all examined species. Notably, *Holstein cow* milk contains significantly higher levels of phosphatidylethanolamine (PE). Distinct phospholipid profiles emerged between species: *yak* milk demonstrated significantly higher concentrations of sphingomyelin (SM), lysophosphatidylethanolamine (LPE), dimethylphosphatidylethanolamine (dMePE), and bis-methylphosphatidic acid (BisMePA), whereas *buffalo* milk showed preferential accumulation of phosphatidylinositol (PI), phosphatidylserine (PS), phosphatidylglycerol (PG), and lysophosphatidylcholine (LPC). Longitudinal analysis revealed dynamic changes in *yak* milk phospholipids during lactation: as the lactation period in-creases, PC, PS, LPC, LPE, methylphosphatidylcholine (MePC), BisMePA, and dMePE exhibited progressive decline, while PE, SM, PI and PG showed incremental increases. Analysis of phospholipid metabolism pathways indicates that *yak* colostrum supports early calf development by enriching phospholipids associated with immune and neuroprotection, while mature milk shifts toward maintaining membrane stability. These compositional characteristics position *yak* milk as a promising phospholipid-fortified alternative to human breast milk.

## 1. Introduction

Milk is a complex oil-in-water emulsion containing essential components such as proteins, lipids, carbohydrates, vitamins, minerals, and bioactive substances, which provide infants with comprehensive nutrition and immune protection [1]. The concentration of milk’s main constituents varies among species, reflecting different energy needs and growth rates of newborns [2]. Cow milk constitutes about 80% of global production, whereas milk from *buffalo* and *yak* holds notable nutritional and economic value in certain regions. As milk’s research significance in scientific, technological, and health-nutrition fields grows, consumer interest has shifted from traditional nutrition to precision and personalized health, increasing demand for more nutritious options. Milk from minor dairy animals such as *buffalo* and *yak* is highly nutritious [3,4], making their products widely popular in China [5].

Milk fat is a valued, widely consumed nutrient, acting as a key energy source and a reservoir of essential fatty acids and vitamins [2,6]. About 98% of milk fat exists as milk fat globules (MFG), each enveloped by a three-layered membrane secreted by mammary epithelial cells—the milk fat globule membrane (MFGM) [7]. Originating from the endoplasmic reticulum and apical plasma membrane of mammary epithelial cells [8], its core components include phospholipids, sphingolipids, and various functional proteins such as BTN and MUC1 [9]. Lipids within MFGM can be categorized into polar lipids and neutral lipids. The primary polar lipids across all mammalian species include phosphatidylethanolamine (PE), phosphatidylcholine (PC), phosphatidylserine (PS), phosphatidylinositol (PI), lysophosphatidylcholine (LPC), and sphingomyelin (SM). The neutral lipids in MFGM primarily consist of triglycerides, with palmitic acid and oleic acid being the predominant fatty acids [10]. Recent studies have confirmed that methylated phospholipids, a low-abundance lipid class, are widely distributed throughout the entire cell membrane system and participate in regulating membrane fluidity and signal transduction [11]. Methylated phospholipids include monomethylphosphatidylcholine (MePC), dimethylphosphatidylethanolamine (dMePE), and bis-methylphosphatidic acid (BisMePA). MFGM also includes proteins, glycoproteins, enzymes, and cholesterol. Several components play crucial roles in human physiology, such as promoting immune and neural development and offering antibacterial, anticancer, and antitumor benefits [12]. Phospholipids aid in infant brain, nervous, and digestive system development and improve cognition [13]. MFGM proteins inhibit breast cancer cell growth, suppress pathogens like *Staphylococcus aureus*, *Escherichia coli*, and *Salmonella* in infant guts, and combat rotavirus [14]. Thus, MFGM is an excellent functional ingredient for infant formula and other functional foods. Moreover, due to phospholipids’ amphiphilic nature, MFGM can serve as an emulsifier, stabilizer, or encapsulation material, maintaining ingredient stability in various liquids [15].

Recently, lipidomics has become a prevalent approach for characterizing complex lipid profiles, including milk fat’s molecular diversity, fine structure, and accurate quantification [16]. Sprenger et al. performed a detailed lipid analysis of three MFGM-rich whey concentrates, detecting 23 lipid subclasses and 5714 lipid molecules [17]. *Holstein cow* are the most bred and highest-yielding dairy breed globally, with their milk dominating consumption [18]. *Buffalo* are the second-largest milk source worldwide [19]. *Buffalo* milk contains more protein, lipids, lactose, and minerals than cow’s milk, with nearly double the αs2-casein, κ-casein, and fat content [20,21]. Beyond nutritional superiority, it possesses health benefits like antihypertensive [22], antioxidant [23], anticancer [24], anti-inflammatory [25], and immune-enhancing effects [26]. Pegolo et al. analyzed 272 buffalo milk samples using gas chromatography, identifying 51 fatty acids. In terms of mass percentage, saturated fatty acids constituted the primary component of buffalo milk fat (70.49%), while monounsaturated and polyunsaturated fatty acids accounted for 25.95% and 3.54%, respectively [27]. *Yak* milk is a nutrient-dense “natural concentrate”, rich in fat, protein, lactose, vitamins, minerals, and bioactive peptides, giving it unique physiological functions [4,28]. Studies show highlanders avoid chronic diseases and deficiencies despite no fruit or vegetable intake, underscoring *yak* milk’s dietary importance for Tibetans [29]. Earlier work described *yak* milk’s lipid profile and globule morphology, showing larger [30] average globule size (~4.39 μm) versus *Holstein* milk (~3.87 μm) and higher cholesterol and sphingolipid content [31]. Later, Luo et al. used in vitro infant digestion models to confirm higher lipolysis in *yak* milk than cow milk, possibly due to more free fatty acids and smaller released particles [32]. While MFGM lipids from various sources have been studied, *buffalo* and *yak* MFGM lipidomics remain underexplored, especially regarding phospholipid differences across *Holstein cow*, *buffalo*, and *yak*.

MFGM properties are affected by lactation stage, altitude, season, diet, and health [33]. Studies often define colostrum as <5 days postpartum, transition milk as 5 days–2 weeks, and mature milk as >2 weeks [34]. Total phospholipids generally decrease from colostrum to mature milk, though trends vary among specific phospholipids, reflecting their distinct dynamics during lactation [35]. Late-lactation milk (>200 DIM) shows higher fat and SM content [36], potentially influenced by lower milk yield [37]. Cheema et al. also noted gradual SM and PC increases from early (DIM = 21) to late lactation (DIM = 217) [38]. Lipid differences between colostrum and mature milk indicate physiological adaptation to neonatal nutritional needs [39]. Understanding milk lipid changes and related metabolic pathways during lactation is vital for improving dairy quality and precise nutrition. However, despite lipid analyses in human, bovine, donkey milk, and formula across lactation, *yak* MFGM phospholipid changes during lactation remain unstudied.

This study explored variations in MFGM phospholipids among colostrum from different bovine species, and a particular focus on the phospholipid profiles of *yak* colostrum and mature milk. First, *yak* milk as well as *Holstein* and *buffalo* milk were collected from Gansu, Beijing, and Guangxi in China, respectively. Subsequently, MFGM were separated and extracted from different milks, and lipids were further extracted. Finally, differential phospholipids were identified and screened by UHPLC-MS/MS combined with whole-target lipidomics. This study is essential to reveal the phospholipid composition of MFGM from different bovine colostrum and from *yak* milk at different stages of lactation. These findings provide a scientific foundation for utilizing MFGM as a functional food or food ingredient.

## 2. Materials and Methods

### 2.1. Materials

LC-MS grade isopropanol (IPA), methanol (MeOH) and acetonitrile (ACN) were purchased from Fisher Scientific (Loughborough, UK). Chloroform was purchased from Sinopharm (Shanghai, China). Formic acid was purchased from TCI (Shanghai, China). Ammonium formate was purchased from Sigma-Aldrich (Shanghai, China). Sodium chloride (NaCl), potassium chloride (KCl), disodium hydrogen phosphate (Na_2_HPO_4_), and potassium dihydrogen phosphate (KH_2_PO_4_) (analytical grade) were purchased from G Protein (Beijing, China). Ultrapure water was prepared using a Milli-Q system (Millipore, Bedford, MA, USA).

### 2.2. Raw Milk Sample Collection

In this study, we manually collected samples from all animals confirmed to be disease-free through screening, with parity ranging from 2 to 4. All milk samples were collected in June 2023. *Holstein* colostrum samples from three adult *Holstein cows* were collected within 0–3 days postpartum at a dairy farm in Daxing District, Beijing. Concurrently, *Buffalo* colostrum samples from three adult *Nile buffaloes* were collected within 0–3 days postpartum at the Guangxi Water *Buffalo* Research Institute of the Chinese Academy of Agricultural Sciences. *Yak* milk samples were collected from six healthy at herders’ homes on Meiren Grassland in Gannan Tibetan Autonomous Prefecture, China. Three *yak* colostrum samples were collected within 0–3 days postpartum, and three mature *yak* milk samples were collected 2 months postpartum. All grazed on the same pasture for natural feeding. After milking, samples were filtered through disposable gauze into sterile cryogenic tubes, immediately frozen in liquid nitrogen tanks, transported to the laboratory, and stored at −80 °C pending analysis. Each sample was tested individually without pooling.

### 2.3. Separation and Extraction of MFGM

MFGM from different raw milks were extracted according to the method used by Wang et al. with slight modifications [40]. The collected milk samples were ultracentrifuged at 4 °C, 10,000× *g* for 30 min, and the upper fat layer was collected. Then the fat layer was washed with PBS, and the centrifugation washing was repeated 3–4 times. The fat was then placed in a 4 °C environment for 12 h to promote hydration. Subsequently, it was subjected to sonication at 25 Hz for 10 cycles of 15 s each. Finally, the sample was centrifuged at 20,000× *g* for 60 min at 4 °C, and the MFGM pellet was collected.

### 2.4. Extraction of Lipids from MFGM

Extraction of MFGM lipids was performed according to the method used by Werner et al. with slight modifications [41]. A 2 mL sample was taken and 750 μL of mixed solvent (chloroform: methanol, 2:1, *v*/*v*) was added and vortexed and shaken for 30 s. The tube was placed on ice for 40 min, 190 μL of water was added, vortexed and shaken for 30 s, and the incubation was continued on ice for 10 min. Centrifuge at 12,000× *g* for 5 min at 20 °C and take 300 μL of the organic layer. Add 500 μL of mixed solvent (chloroform: methanol, 2:1, *v*/*v*) and vortex and oscillate for 30 s. Centrifuge at 12,000× *g* for 5 min at 20 °C. 400 μL of organic layer was extracted, and the sample was concentrated to dryness under vacuum. The sample was dissolved with 200 μL of isopropanol and the supernatant was filtered through a 0.22 µm filter membrane to obtain a prepared sample for UHPLC-MS/MS analysis.

### 2.5. Determination of MFGM Lipids by UHPLC-MS/MS

Phospholipids in MFGM were determined using a UHPLC-MS/MS system according to the method reported by Chen et al. with slight modifications [42]. In particular, the chromatographic separation was carried out on an ACQUITY UPLC^®^ BEH C18 column (2.1 × 100 mm, 1.7 µm, Waters) under chromatographic conditions, with the column temperature maintained at 50 °C and the autosampler temperature at 8 °C. In addition, gradient elution was performed using acetonitrile/water = 60:40 (containing 0.1% formic acid and 10 mM ammonium formate) (A2) and isopropanol/acetonitrile = 90:10 (containing 0.1% formic acid and 10 mM ammonium formate) (B2) as the mobile phases, at the flow rate of 0.25 mL/min, and the sample was injected into the system after equilibrium for 2 μL. The mass spectrometry conditions were determined according to the ESI-MSn experiments in the positive and negative ion mode, respectively. Spray voltages of 3.5 kV and 2.5 kV were used under ESI-MSn experiments, respectively. The sheath gas and auxiliary gas were set to 30 and 10 arbitrary units, respectively, the capillary temperature was 325 °C, and the Orbitrap analyser was used in full scan mode with a scanning mass range of *m*/*z* 150–2000 and a mass resolution of 35,000. Additionally, the data-dependent acquisition (DDA) MS/MS experiments were performed using the HCD scan mode with a normalized collision energy of 30 eV. The dynamic exclusion function was used to remove unnecessary redundant information from the MS/MS spectra.

### 2.6. Primary Processing of Lipidomics Data

Raw mass spectrometry data after down-conversion were annotated by LipidSearch software (V4.2.28). Next, peak alignment and peak filtering were processed using the following parameters, bR.T. Tolerance = 0.25, m-Score Threshold = 3. The resultant quantitative listings included lipids, mass-to-charge ratios (mass-to-charge ratios, *m*/*z*), retention times (retention times, rt), and peak response values (intensities). Sum peak normalization was performed to allow comparison of data of different orders.

### 2.7. Statistical Analysis

All experiments were performed in three independent operations, statistical analysis was performed using SPSS 23, and *t*-tests were used for data analysis. Orthogonal Partial Least Squares Discriminant Analysis (OPLS-DA) was performed using the R software (rpy2) package and the model was tested for overfitting using the permutation test; lipid molecules were considered statistically significant when *p* < 0.05 and VIP > 1. Correlations between different lipid molecules were analyzed using the Spearman method and the Leiden cluster algorithm, and metabolites in lipid biosynthesis pathways were characterized and identified using commercial databases, including the Kyoto Encyclopedia of Genes and Genomes (KEGG) Pathway Database https://www.kegg.jp/kegg/ (accessed on 29 April 2025), the Human Metabolome Database (HMDB) https://hmdb.ca/ (accessed on 16 May 2025) and the PubChem database https://pubchem.ncbi.nlm.nih.gov (accessed on 18 May 2025).

## 3. Results and Discussion

### 3.1. Clustering Analysis of MFGM Lipid in Yak, Holstein, and Buffalo Colostrums

**Figure 1:**
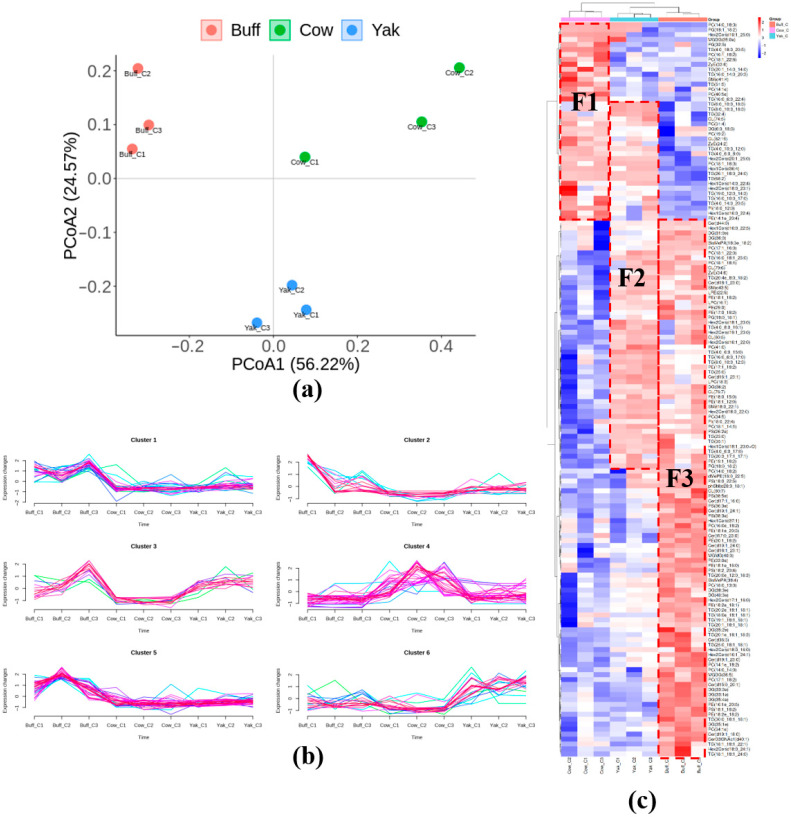
Lipid differences in MFGM of cow’s milk from different species. (**a**) PCA score chart. (**b**) Trend analysis Overall lipid heat map. (**c**) Overall lipid heat map. Here, Buff, Cow, and Yaw denote the lipid composition of MFGM from *buffalo* milk, *Holstein* milk, and *yak* milk sources, respectively.

The lipidomic profiles of MFGM lipid extracts from *Holstein cow*, *yak*, and *buffalo* colostrum samples, along with quality control samples, were examined to evaluate the reproducibility of sample preparation and the reliability of the analytical method. Principal components analysis (PCA) aims to use the idea of dimensionality reduction to transform multiple indicators into a few composite indicators [43]. As shown in Figure 1a, the closer distance of the samples between the same groups indicates a better reproducibility of the samples, while the greater distance between the different groups indicates a greater difference in the lipid composition of the MFGM of the different bovine milk species. They were separated along PCA1 (56.22%) and PCA2 (24.57%). The study shows that a higher contribution rate indicates that the principal component is more important, and a cumulative contribution rate of more than 70% of the principal components means that they can effectively represent the data characteristics [44].

Cluster analysis was performed on MFGM lipids from colostrum of three bovine species. As shown in Figure 1c, there were significant differences in the lipid composition of MFGM in *Holstein cow*, *yak*, and *buffalo* colostrum. Highly expressed lipids were selected as representative lipids, and the representative lipids were F1, F2 and F3, containing 40, 50 and 58 lipids, respectively, of which 24 common lipids were present in F1 and F2, and 26 common lipids were present in F2 and F3. From the perspective of biological evolution and nutrition, *Holstein cows* were originally bred in temperate environments and are characterized by high milk production, lower fat content, and higher protein levels—traits resulting from artificial selection. From a genetic perspective, high milk yield is typically associated with lower milk components [45]. The *yak* adapts to the extreme cold, low oxygen, and strong ultraviolet environment of the Qinghai–Tibet Plateau, and the milk composition is obviously high fat and high protein, which is the result of strong natural selection to ensure the survival rate of offspring in the harsh environment [46]. In addition, *buffalo* is adapted to tropical/subtropical, hot and humid environments, and high fat is the most prominent feature of *buffalo* milk [47]. Both *yaks* and *buffaloes* have evolved high-fat milk in extreme environments, a convergent adaptation to the need to ensure high survival of offspring under the pressures of their respective environments. The MFGM lipids in the colostrum of *yak* milk contain the common components of *buffalo* milk and *Holstein* milk, which may be more nutritionally advantageous when used as the base material of infant formula.

### 3.2. Lipid Expression Trend Analysis

Clustering of phospholipid compounds via multivariate statistical analysis. Each cluster in the figure represents a group of phospholipid compounds exhibiting similar expression trends, meaning their relative abundance across different samples follows consistent patterns. The results of further trend analysis are shown in Figure 1b, in which the lipids in the MFGM of *buffalo* colostrum were mainly highly expressed in clusters 1 and 5, *Holstein cows* mainly highly expressed lipids in cluster 4, while *yak* milk was mainly concentrated in cluster 6. Among them, Cluster 1 mainly contains BisMePA(18:3e_18:2), CL(79:6), CL(80:7), Cer(t18:1_23:1), Cer(t19:1_18:0), Cer(t19:1_23:0), Cer(t19:1_24:0), Cer(t19:1_24:1). Cer(t17:0_23:0), Hex1Cer(d16:0_22:5), Hex1Cer(d37:1), Hex2Cer(d16:1_24:1), Hex2Cer(d17:1_16:0), MGMG(40:0), PC(14:0_14:0), PC(14:0_18:2), PC(17:1_16:0), PC(17:1_18:2), PE(17:0_18:2), PE(18:2e_18:1), PE(18:2e_18:2), PS(18:1_18:2), PS(18:2_20:4), PS(38:5e), TG(20:3_17:1_17:1). ZyE(34:6), and dMePE(16:0_22:5). Whereas the lipids in cluster 4 are mainly CL(82:19), DG(6:0_18:3), Hex1Cer(d14:0_22:4), Hex1Cer(d16:0_22:4), Hex1Cer(d36:4), Hex2Cer(d18:0_23:1), Hex2Cer(d19:1_25:0), the Hex2Cer(d20:1_25:0), PC(14:0_18:3), PC(14:1e), PC(16:1_18:2), PC(18:1_18:3), PC(18:1_22:6), PC(19:2), PC(31:4), PC(40:5e), PE(14:1e_20:4). PG(18:1_ 18:2) PG(32:5), PI(18:0_12:0), SM(d41:4), TG(16:0_10:0_17:0), TG(16:0_14:0_20:3), TG(16:0_6:0_22:4), TG(19:0_12:0_14:3), TG(20:1_14:0_14:0), TG(26:1_18:0_24:0), TG(4:0_10:3_12:0), TG(4:0_14:0_20:5), TG(4:0_18:0_20:5), TG(4:0_6:0_8:0), TG(51:5), TG(68:2), ZyE(24:2) and ZyE(33:6). Notably, the main concentration of cluster 6 highly expressed lipids in *yak* milk was mainly CL(74:5), Cer(d16:1_23:1), Hex2Cer(d18:1_23:0), LPC(18:3), LPE(22:5), MGDG(35:0e), PC(18:1_14:1), PC(18:1_22:0), PC(34:5), PC(41:0), PE(17:1_18:2), PG(18:0_18:2), TG(16:0_18:1_23:0), TG(16:0_6:0_17:0), TG(25:0), TG(32:4), TG(4:0_6:0_15:0), TG(4:0_8:0_ 16:1), TG(6:0_10:0_18:3), TG(8:0_10:0_12:3), and TG(8:0_10:3_18:3).

Milk fat globules are predominantly composed of triglycerides (TGs, ~98% of total lipids), with minor fractions of polar lipids and cholesterol. Among these, phospholipids (PLs) and sphingolipids (SLs) represent the primary polar lipid classes. Although polar lipids constitute only 0.2–1.0% of total milk fat, they account for 26–40% of the MFGM [48].The MFGM-associated polar lipids exhibit a diverse profile, including phosphatidylcholine (PC; 35%), phosphatidylethanolamine (PE; 30%), sphingomyelin (SM; 25%), phosphatidylinositol (PI; 5%), phosphatidylserine (PS; 3%), as well as glucosylceramide (GluCer), lactosylceramide (LacCer), and trace gangliosides [49]. Comparative lipidomic analyses reveal significant interspecies variation in MFGM composition. For example, Sun et al. identified 73–156 differentially abundant lipids across human, bovine, and caprine milk, spanning 11 lipid classes: PC, PE, SM, phosphatidylglycerol (PG), PI, PS, ceramide (Cer), phosphatidic acid (PA), hexosylceramide (HexCer), dihexosylceramide (Hex2Cer), and cardiolipin (CL) [10]. Further evidence of compositional divergence comes from He et al. (2024), who detected 353 distinct lipid species in human and camel milk MFGMs, including 77 PEs, 30 PCs, 28 PIs, 59 SMs, 54 Cers, 20 PSs, 4 PGs, 13 lysophosphatidylcholines (LPCs), and 14 lysophosphatidylethanolamines (LPEs) [49]. These findings underscore substantial heterogeneity in MFGM lipid profiles across mammalian species.

### 3.3. Lipid Distribution in MFGM of Different Colostrums

**Figure 2:**
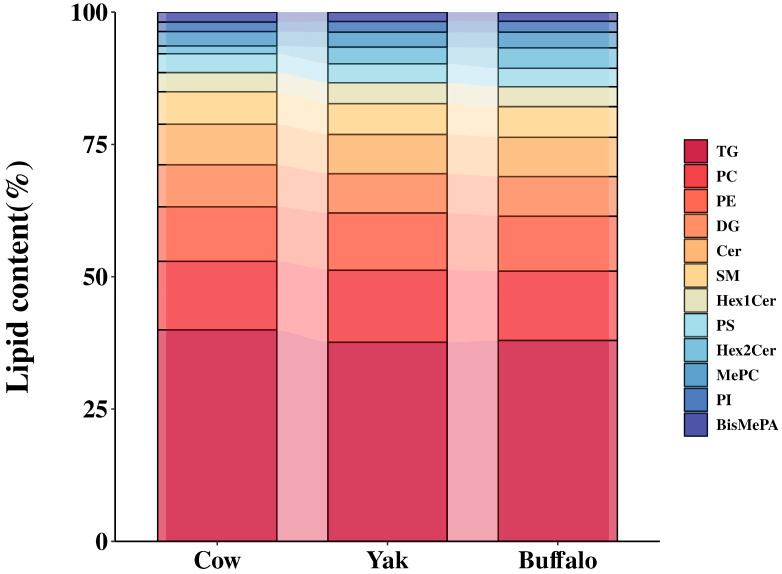
Classification of lipid content in the top 12 positions across three groups of colostrum samples.

A total of 1739 lipid molecules were identified in MFGM from *Holstein* colostrum, *buffalo* colostrum, and *yak* colostrum. The identified lipids were systematically classified based on fatty acid chain length, molecular composition, and structural characteristics (Figure 2), encompassing 43 distinct subclasses. The dominant lipid species included triglycerides (TG), phosphatidylcholine (PC), phosphatidylethanolamine (PE), diacylglycerol (DG), ceramides (Cer), sphingomyelin (SM), dihexosylceramide (Hex2Cer), monohexosylceramide (Hex1Cer), phosphatidylserine (PS), and phosphatidylinositol (PI). Comparative analysis of the top 12 lipid subclasses across three groups of colostrum samples (Figure 2) revealed that variations predominantly stemmed from quantitative differences rather than qualitative distinctions in lipid categories. TG emerged as the predominant lipid fraction, constituting over 39% of total lipids. Meanwhile, phospholipids (PLs) and sphingolipids (SLs) were also quantitatively significant, with PC and PE representing the most abundant subclasses. These observations align with prior findings [10]. Notably, PC, PE and SM—the primary polar lipids in the MFGM—exhibit concentration variations attributable to species-specific and dietary factors [50]. Beyond their structural role in membrane organization, these lipids enhance nutritional functionality [19]. For instance, Phosphatidylcholine (PC), as the predominant phospholipid (PL) constituent, serves as a critical biosynthetic precursor for plasma membrane PLs. Its enzymatic hydrolysis yields acetylcholine, a key neurotransmitter essential for synaptic signaling. Phosphatidylethanolamine (PE) plays a multifunctional role in cellular homeostasis, regulating processes such as intracellular signaling cascades and programmed cell death [51]. Among sphingolipids (SLs), sphingomyelin (SM) dominates the MFGM lipidome across mammalian species. Beyond its structural role in myelin sheath formation, SM modulates neuronal excitability and axonal conduction efficiency, directly influencing pediatric cognitive performance [52]. Evidence indicates that infants receiving SM nutritional interventions exhibit positive correlations with neurobehavioral development characterized by higher language development levels [53]. These neurodevelopmental benefits underscore SM’s functional significance in early-life nutrition.

Furthermore, the MFGM sphingolipid (SL) fraction contains biologically significant constituents, including ceramide (Cer), monohexyl ceramide (Hex1Cer), and dihexosylceramide (Hex2Cer), which contribute to neonatal neural development and modulate neutral lipid metabolism. Previous studies have primarily characterized the MFGM lipidome, identifying major subclasses such as triglycerides (TG), diacylglycerol (DG), glycerophospholipids (e.g., PC, PE, PG, PA, PS, PI, CL), and sphingolipids (e.g., SM, Cer, Hex1Cer, Hex2Cer) [54]. Notably, the present study reveals previously unreported methylated phospholipids—bis-methylphosphatidic acid (BisMePA), methylphosphatidylcholine (MePC), and dimethylphosphatidylethanolamine (dMePE)—in colostral MFGM, thereby expanding its known lipidomic diversity. These methylated phospholipid derivatives play crucial structural and functional roles, particularly in membrane stabilization and phospholipid methylation processes [55].

### 3.4. Differential MFGM Phospholipids in Different Species of Colostrum

**Figure 3:**
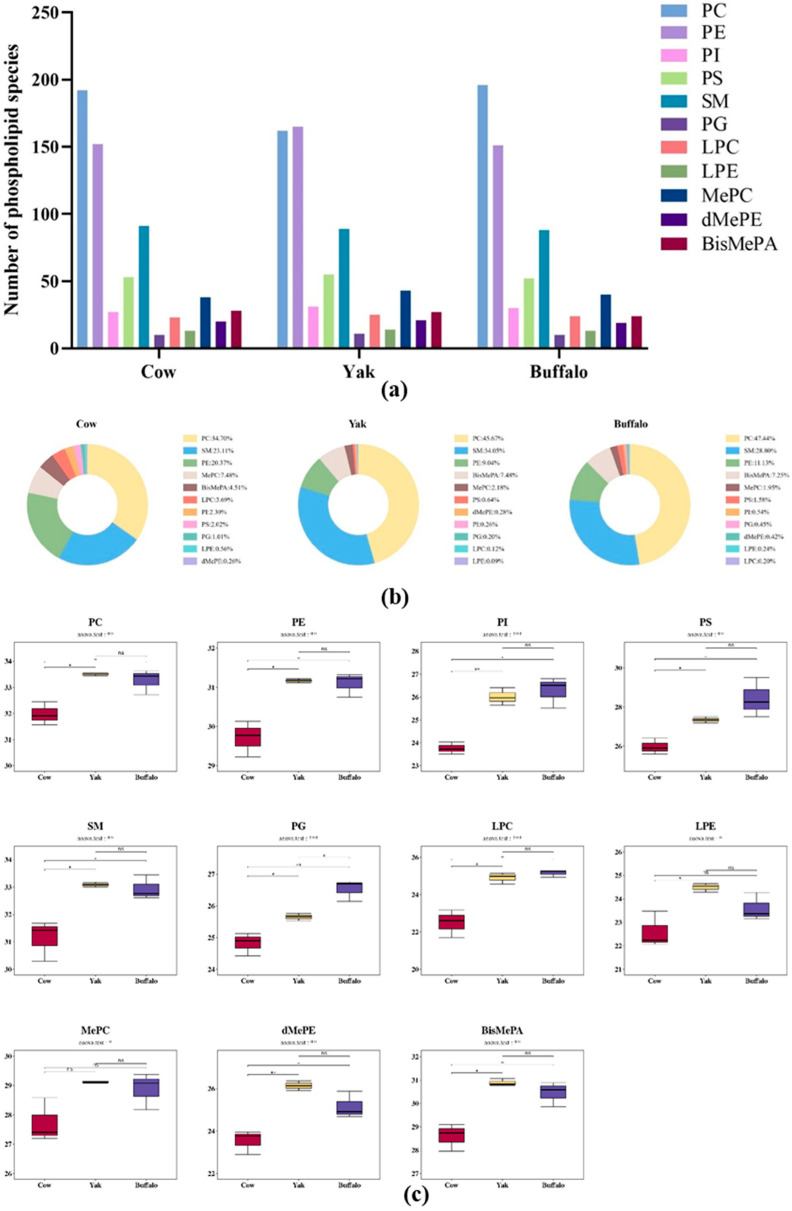
Analysis of differences in MFGM phospholipids in *yak* milk at different stages of lactation. (**a**) Comparison of phospholipid subclass numbers. (**b**) Comparison of the percentage composition of phospholipid subclasses. (**c**) Comparative analysis of phospholipid subclass content.

To characterize the differences in MFGM phospholipids in different bovine species, the types and contents of phospholipids in colostrum of *Holstein cows*, and *buffaloes* were further identified by LC/MS. A total of 710 phospholipid molecules were identified, which could be classified into 11 lipid subclasses, including 25 lysophosphatidylcholine (LPC), 14 lysophosphatidylethanolamine (LPE), 213 phosphatidylcholine (PC), 172 phosphatidylethanolamine (PE), 11 phosphatidylglycerol (PG), 32 phosphatidylinositol (PI), 55 phosphatidylserine (PS), 91 sphingomyelin (SM), 48 methylated phosphatidylethanolamine (MePC), 28 bis-methylphosphatidic acid (BisMePA), and 21 dimethylphosphatidylethanolamine (dMePE). Figure 3a illustrates the detected quantities of each phospholipid subclass in MFGM from *Holstein cows*, *buffalo*, and *yak* colostrum. The most molecularly diverse PLs in the three colostrums were PC (160–200 species) and PE (150–170 species). Figure 3b shows the relative content of each subclass of PL in *Holstein* milk, *yak* milk and *buffalo* colostrum. The highest percentage of phospholipids being PC (34.7% in *Holstein* milk, 45.67% in *yak* milk and 47.44% in *buffalo* milk), followed by SM (23.11% in *Holstein* milk, 34.05% in *yak* milk and 28.80% in *buffalo* milk) and PE (20.37% for *Holstein* milk, 9.04% for *yak* milk and 11.13% for *buffalo* milk), which shows that PC, SM and PE are the three phospholipid subclasses that accounted for a larger proportion of the total. PC was the most abundant phospholipid (34.7% to 47.44%) in all cattle species, consistent with its core structural function in biofilms [56]. Meanwhile, the higher proportion of PE in the milk of *Holstein cows* may be associated with efficient lactation, and PE promotes membrane fusion and splitting and facilitates milk fat globule formation [51]. The advantage of PE in *Holstein cows*, which have been selected for a long period of time, may accelerate milk fat globule secretion and enhance milk production efficiency.

The contents of the 11 phospholipids were transformed by log 2 and finally represented as box plots in Figure 3c. The results showed that *yaks* had the highest content of SM, LPE, dMePE, and BisMePA. dMePE and BisMePA, which are methylated phospholipids (PL) components, play a crucial role in maintaining the intermolecular structure of membranes [55]. *Buffaloes* have the highest levels of PI, PS, PG, and LPC, and *buffaloes* live in high-temperature and high-humidity environments, where PI may regulate mammary cell stress response to high temperatures by enhancing intracellular metabolic signaling [57]. Meanwhile, high levels of LPC provide natural antimicrobial properties in *buffalo* [58]. Notably, both species contain significantly higher levels of all phospholipid subclasses, including PC and PE, compared to *Holstein* milk, with no significant difference between the two. This may be attributed to their respective adaptations—*Yak* and water *buffalo* stabilize milk fat globules by enhancing phospholipid synthesis in the mammary membrane to cope with extreme temperatures.

### 3.5. Differences in MFGM Lipids in Yak Milk at Different Stages of Lactation

**Figure 4:**
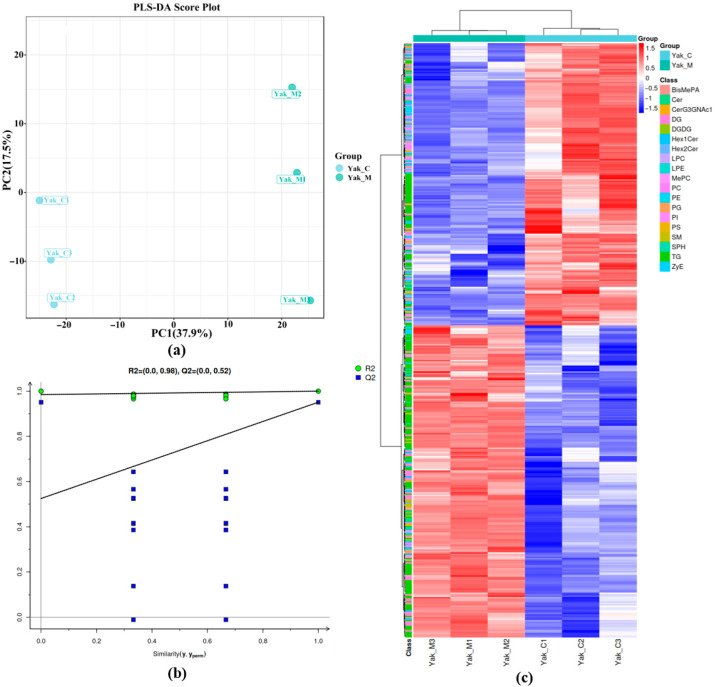
Differential analysis of MFGM phospholipids in *yak* milk at different lactation stages. (**a**) PLA-DA analysis. (**b**) PLS-DA replacement test (**c**) Analysis of differences in lipid metabolism between the two groups.

To achieve a higher level of group separation, Partial Least Squares Discriminant Analysis (PLS-DA) was used to analyze the variables responsible for classification. As shown in Figure 4a, samples of MFGM lipids from *yaks* of different lactation stages were close together. This indicates that there is a significant difference in the lipid composition of MFGM and their respective lipids in the milk of cows of different lactation stages, whereas the closer distance of the samples in the same group indicates better reproducibility. In addition, it was also effective in predicting differences in lipid composition between groups, with R^2^ = 0.98 and Q^2^ = 0.52 (Figure 4b). By randomly changing the order of the distribution variable Y 200 times and building the corresponding PLS-DA model, values of R^2^ and Q^2^ can be obtained, which are used for detecting overfitting. An R^2^ closer to 1 indicates a more stable model, and a Q^2^ > 0.5 indicates a high prediction rate, suggesting that the model has excellent discriminatory power and no risk of overfitting. Overall, there were significant differences between any two groups, suggesting that the PLS-DA model can be used to identify differences between the MFGM of milk at different lactation stages. Heat map cluster analysis was plotted to further demonstrate the overall differences in the lipids on the MFGM of cow’s milk in different lactations, and it can be seen that there are large differences between lactations (Figure 4c).

### 3.6. Differences in MFGM Phospholipids in Yak Milk at Different Stages of Lactation

**Figure 5:**
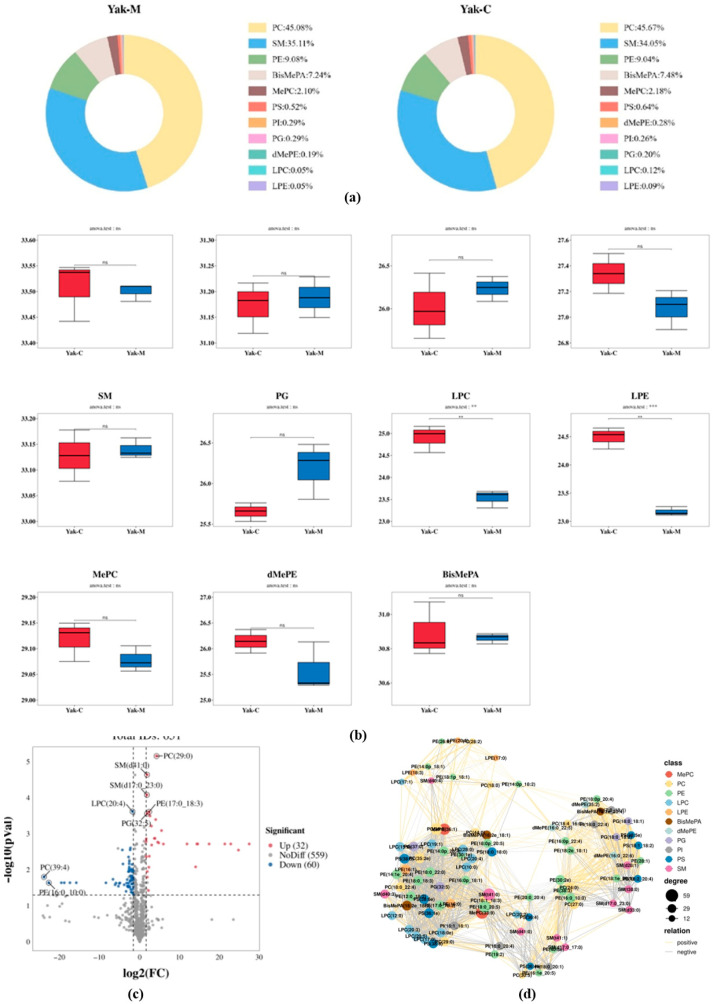
Differential analysis of MFGM phospholipids in *yak* milk at different lactation stages. (**a**) Comparison of the percentage composition of phospholipid subclasses between *yak* colostrum and mature milk. (**b**) Comparison of the concentration of each phospholipid subclass between *yak* colostrum and normal milk. (**c**) Volcanogram of differential lipids between *yak* colostrum and mature milk. (**d**) Differential lipid metabolism correlation network diagram.

A total of 651 phospholipid molecules were identified in *yak* colostrum and mature milk samples, which could be classified into 11 lipid subclasses, including 25 lysophosphatidylcholine (LPC), 14 lysophosphatidylethanolamine (LPE), 162 phosphatidylcholine (PC), 168 phosphatidylethanolamine (PE), 11 phosphatidylglycerol (PG), 33 phosphatidylinositol (PI), 55 phosphatidylserine (PS), 90 sphingomyelin (SM), 44 methylated phosphatidylethanolamine (MePE), 28 bis-methylphosphatidic acid (BisMePA), and 21 dimethylphosphatidylethanolamine (dMePE). Thus, PC, PE, and SM determined the abundance of MFGM phospholipids in *yak*. Meanwhile, in addition to the previously reported phospholipids found in *yak* MFGM, MePC, BisMePA, and dMePE were additionally found in the present study, which provided a basis for enriching the variety of *yak* MFGM phospholipid composition. The total concentration of each phospholipid subclass is the sum of the phospholipid concentration of that class. As shown in Figure 5a, the phospholipids with the highest percentage in both colostrum and mature milk of *yaks* were PC (45.67% in colostrum and 45.08% in mature milk), followed by SM (34.05% in colostrum and 35.11% in mature milk) and PE (9.04% in colostrum and 9.08% in mature milk).

Furthermore, considering that the proportion of each phospholipid subclass in milk changes with lactation as the calf’s nutritional requirements change during growth, we explored the trend changes in the concentration of each phospholipid subclass at different stages of lactation (Figure 5b). It is worth noting that the screening criteria for differentiated lipids between *yak* colostrum and *yak* mature milk were based on variable weight values (VIP > 1.0), *t*-tests (*p* value < 0.05), and fold change (FC > 3) of the PLS-DA model. A total of 92 significantly different phospholipids were screened in *yak* colostrum and mature milk, including 12 PCs, 26 PEs, 5 PIs, 10 PSs, 10 SMs, 4 PGs, 12 LPCs, 5 LPEs, 2 MePCs, 3 dMePEs, and 3 BisMePAs (Figure 5c). A total of 32 phospholipids were significantly up-regulated and 60 phospholipids were significantly down-regulated in mature milk. Notably, PC (29:0), SM (d41:0), SM (d17:0_23:0), LPC (20:4), PE (17:0_18:3), PG (32:5), PC (39:4), and PE (16:0_10:0) differed significantly between *yak* colostrum and mature milk, which is an important for distinguishing between different lactation stages and can serve as their contribution and may be used as potential biomarkers for their corresponding milk.

With prolonged lactation, the levels of PC, PS, LPC, LPE, MePC, BisMePA, and dMePE decrease, and the levels of PE, SM, PI, and PG increase. PC, SM, and PE are the three more predominant phospholipid subclasses, and PC is a highly abundant precursor of plasma membrane PL and is the most prevalent component of PL. It produces acetylcholine, an important neurotransmitter [59]. In addition, PE is essential in physiological processes such as signal transduction and apoptosis [51]. It also promotes brain function and enhances memory [60]. SM is mainly produced by transferring phosphorylcholine groups from PC to ceramide via sphingomyelin synthase [61]. Therefore, the significant decrease in PC content during lactation may be due to its partial conversion to SM during metabolism, resulting in a gradual increase in SM content and proportion. SM is involved in the formation of MFGM lipid rafts and neuromyelin sheaths in the brain, and thus SM has a role in promoting neurodevelopment in infants [31]. The increasing trend of SM content during lactation reflects the early neurological development of infants. Therefore, special infant formulas, such as preterm infants, should be supplemented with SM appropriately.

### 3.7. Enrichment Analysis of Differential Phospholipid-Related Networks and Metabolic Pathways in Yak Colostrum and Mature Milk

Most lipids showed large correlations, and correlations were usually stronger for the same phospholipid species than between different phospholipid species (Figure 5c). It was proposed in earlier studies that changes in the content of individual lipids at different lactation stages may have a significant effect on the metabolic expression of other lipids. The correlation between different PL subclasses may be due to the exchange of PL first groups [62]. PS can be decarboxylated to generate PE catalyzed by phosphatidylserine decarboxylase (PSD) [63]. LPE, the lysophosphatidic form of PE, may be reacylated to generate PE by acyltransferases such as LPEAT [51]. Elevated PE levels alongside decreased PS and LPE in mature milk suggest decarboxylation and re-esterification may occur during lactation to maintain membrane integrity. PC generates SM by transferring phosphorylcholine groups to ceramides via sphingomyelin synthase (SMS). The high correlation between PC and SM further suggests that an increase in SM in the late lactation period may deplete PC, leading to a decrease in PC content [64]. In addition, PC can also generate PS by replacing choline with serine via phosphatidylcholine/serine exchange enzyme (PSS). The decrease in PC accompanied by a decline in PS in late lactation may be due to a shift in mammary cells to other metabolic demands (e.g., SM synthesis), resulting in a decrease in the conversion of PC to PS [63]. Thus, PS is highly correlated with PC and PE in the correlation network. With the decrease in PC content in lactation, PS content was also affected, which may be one of the reasons for the decrease in PS content in lactation. Suggesting that hypermethylated phospholipids in colostrum may support immunoreactivity by regulating membrane charge or signaling, whereas mature milk is more focused on the stability of the underlying membrane structure. The correlation between the same subclasses of PLs may be due to the same synthetic pathway as well as the hydrolysis and remodeling of PLs [65,66]. The screening and correlation network analysis of significantly different PLs laid the foundation for studying the regulation and function of PLs.

To investigate the metabolic pathways of differential phospholipids between *yak* colostrum and mature milk, the significantly different phospholipids were mapped to the KEGG database for information. The results showed that colostrum preferentially supports immune establishment and neuroprotection in calves through high expression of PS, acetylated phospholipids and PC. In contrast, mature milk focuses on the homeostatic maintenance of PE and SM to safeguard membrane function for sustained lactation as well as neurodevelopment and intestinal health of calves. This study provides a molecular basis for analyzing the natural nutritional advantages of *yak* milk and lays a theoretical foundation for the development of functional MFGM products, such as immune enhancers or antioxidant formulations.

## 4. Conclusions

This study investigated variations in phospholipids within the MFGM across different bovine species and lactation stages. The results showed that PC was the most abundant phospholipid in MFGM (34.7–47.44%) in the milk of *Holstein cows*, *yaks* and *buffaloes*, and the higher proportion of PE in the milk of *Holstein cows* might be related to efficient lactation. *Yaks* had the highest content of SM, LPE, dMePE, and BisMePA, and *buffaloes* had the highest content of PI, PS, PG, and LPC, suggesting that there is a tendency for convergent evolution of MFGM phospholipids in *yaks* and *buffaloes*. In addition, the levels of PC, PS, LPC, LPE, MePC, BisMePA, and dMePE in *yak* milk decreased and the levels of PE, SM, PI and PG increased with the prolongation of lactation. In this study, the presence of MePC, BisMePA and dMePE was found in *yak* milk for the first time. High expression of PC, PE and lysophosphatidic phospholipids (LPC, LPE) in *yak* colostrum supports biofilm composition, cognitive development and lipid metabolism, whereas high expression of SM and PS in mature milk enhances membrane stability and antioxidant capacity and participates in neuromyelin formation in the brain. This suggests an advantage of *yak* milk as a substitute for breast milk in terms of phospholipid trends.

## Data Availability

Data will be made available on request.
